# A real-world comparison of outcomes between fractional flow reserve-guided versus angiography-guided percutaneous coronary intervention

**DOI:** 10.1371/journal.pone.0259662

**Published:** 2021-12-16

**Authors:** Christopher C. Y. Wong, Austin C. C. Ng, Cuneyt Ada, Vincent Chow, William F. Fearon, Martin K. C. Ng, Leonard Kritharides, Andy S. C. Yong

**Affiliations:** 1 Department of Cardiology, Concord Repatriation General Hospital, Sydney, NSW, Australia; 2 Stanford University School of Medicine, Stanford, CA, United States of America; 3 Department of Cardiology, Royal Prince Alfred Hospital, Sydney, NSW, Australia; University of Massachusetts Medical School, UNITED STATES

## Abstract

**Background:**

Fractional flow reserve (FFR)-guided percutaneous coronary intervention (PCI) has been shown to be superior to angiography-guided PCI in randomized controlled studies. However, real-world data on the use and outcomes of FFR-guided PCI remain limited. Thus, we investigated the outcomes of patients undergoing FFR-guided PCI compared to angiography-guided PCI in a large, state-wide unselected cohort.

**Methods and results:**

All patients undergoing PCI between June 2017 and June 2018 in New South Wales, Australia, were included. The cohort was stratified into the FFR-guided group when concomitant FFR was performed, and the angiography-guided group when no FFR was performed. The primary outcome was a combined endpoint of death or myocardial infarction (MI). Secondary outcomes included all-cause death, cardiovascular (CVS) death, and MI. The cohort comprised 10,304 patients, of which 542 (5%) underwent FFR-guided PCI. During a mean follow-up of 12±4 months, the FFR-guided PCI group had reduced occurrence of the primary outcome (hazard ratio [HR] 0.34, 95% confidence intervals [CI] 0.20–0.56, P<0.001), all-cause death (HR 0.18, 95% CI 0.07–0.47, P = 0.001), CVS death (HR 0.21, 95% CI 0.07–0.66, P = 0.01), and MI (HR 0.46, 95% CI 0.25–0.84, P = 0.01) compared to the angiography-guided PCI group. Multivariable Cox regression analysis showed FFR-guidance to be an independent predictor of the primary outcome (HR 0.45, 95% CI 0.27–0.75, P = 0.002), all-cause death (HR 0.22, 95% CI 0.08–0.59, P = 0.003), and CVS death (HR 0.27, 95% CI 0.09–0.83, P = 0.02).

**Conclusions:**

In this real-world study of patients undergoing PCI, FFR-guidance was associated with lower rates of the primary outcome of death or MI, as well as the secondary outcomes of all-cause death and CVS death.

## Introduction

The development of fractional flow reserve (FFR) has improved the assessment and treatment of coronary artery disease in the catheter laboratory. The DEFER (Deferral Versus Performance of PCI of Non-Ischaemia-Producing Stenoses) and DEFER-DES (Proper Fractional Flow Reserve Criteria for Intermediate Lesions in the Era of Drug-Eluting Stent) studies found that coronary lesions with an FFR value ≥ 0.75 can be safely deferred and medically managed [1, 2], while the FAME (Fractional Flow Reserve Versus Angiography for Multivessel Evaluation) study demonstrated superiority of FFR-guided PCI over angiography-guided PCI in reducing the combined endpoint of death, myocardial infarction (MI), and repeat revascularization [3].

Despite the benefits of FFR-guidance, this technique has not achieved widespread adoption amongst the general interventional cardiology community, with FFR utilized in only a minority of patients undergoing PCI for stable coronary disease [4, 5]. Furthermore, real-world studies comparing outcomes following FFR-guided PCI vs angiography-guided PCI have focused primarily on the US and European population, with no data existing outside of these geographic regions. We therefore performed an analysis on data obtained from a state-wide unselected Australian population to examine clinical outcomes related to FFR-guided PCI in a contemporary PCI cohort.

## Materials and methods

### Data sources

Data for the present study were obtained from the Admission Patient Data Collection (APDC) registry held by the Centre-for-Health-Record-Linkage, one of the largest data linkage systems in Australia. The registry captures health data for patients admitted to ≥ 97% of all health care facilities in the state of New South Wales (NSW), which consists of a population of 7.5 million people. Studies based on this registry have been published previously [6, 7]. The primary and secondary diagnoses associated with each admission, coded according to the International Classification of Diseases Tenth Revision Australian Modification, were extracted from the ADPC registry (S1 Table).

A high-volume public hospital site was defined as one that performed a higher number of PCI than the median of the cohort. All private hospitals were considered as one separate facility as case-volumes for individual private hospitals were not available. The initial cohort comprised all index cases of PCI, coded under the Australian Classification of Health Interventions system (S1 Table), that were performed after 26^th^ June 2017, which marked the date when FFR data was first recorded into the APDC registry. To allow for a pre-specified minimum of six months follow-up for all patients, the study cohort was limited to admissions between 26^th^ June 2017 and 30^th^ June 2018, with 31^st^ December 2018 designated as the end date for study follow-up. The study cohort was subsequently stratified into the FFR-guided PCI group when a concomitant FFR procedure was performed during admission, and the angiography-guided PCI group when no FFR was performed (S1 Fig).

The study protocol conformed to the ethical guidelines of the 1975 Declaration of Helsinki. Approval was granted by the NSW Population and Health Services Research Ethics Committee, reference number: 2013/09/479. The Ethics Committees granted a waiver of the usual requirement for the consent of the individual to the use of their health information. All patient data were de-identified and analysed anonymously.

### Study outcomes

The primary outcome was a combined endpoint of all-cause death or MI. Secondary outcomes included all-cause death, cardiovascular (CVS) death, and MI. The outcome of all-cause death was tracked using the state-wide death registry, which has a non-capture rate of only 0.6% based on known migration rates [8]. Cases were limited to only NSW state residents to minimize incomplete tracking. To ascertain cause-specific mortality, all death certificates were reviewed by two physicians blinded to the patients’ comorbidities and study group allocation. CVS death was defined as death due to MI, heart failure, stroke, arrhythmias, or cardiogenic shock. Disparities in cause-specific mortality coded by the two reviewers were adjudicated by a third reviewer. The endpoint of MI was tracked to subsequent hospital MI presentations recorded within the APDC registry using linkage analysis. MI was defined a priori as occurring >24 hours after the index PCI in order to exclude periprocedural MIs.

### Statistical analysis

The baseline characteristics of the two groups were compared using the t-test for continuous variables, and Pearson’s chi square test for dichotomous variables. Time to primary and secondary outcome events between groups were compared using the Kaplan-Meier method and log-rank test. Cox proportional hazards regression analysis was used to create multivariable models to determine whether FFR-guidance was an independent predictor of outcomes. The proportional hazards assumption was checked with log-minus-log plots. To account for non-CVS death and all-cause death as competing risk events for CVS death and MI respectively, we initially treated these events as censoring and performed standard cox regression modelling. We then separately performed competing risk analyses using the sub-distribution hazard ratio function (Fine and Gray model) [9]. A secondary analysis was performed using propensity score-matching to balance out differences in baseline characteristics between patients in the FFR-guided and angiography-guided PCI groups. Patients in the FFR-guided and angiography-guided PCI groups were matched 1:1 based on their propensity scores using nearest neighbour matching, and outcomes were compared with Kaplan Meier survival curves. Spearman’s rank correlation coefficient was performed to assess the correlation between hospital PCI case volume and use of FFR-guided PCI, with this analysis restricted only to patients in the public hospital sector, as our database could not distinguish between individual hospitals in the private sector. All analyses were performed using SPSS v24 (IBM, USA). A two-tailed probability value < 0.05 was considered statistically significant.

## Results

### Baseline characteristics

From 26^th^ June 2017 to 30^th^ June 2018, 10,304 PCIs were performed in NSW and formed the basis of the study cohort. FFR-guided PCI was performed in 542 (5%) of cases, while angiography-guided PCI was performed in 9762 (95%) of cases. There were no significant differences in age, gender, comorbidities, and multi-vessel PCI between the FFR-guided and angiography-guided group. Patients in the FFR-guided group were significantly less likely to present with acute coronary syndrome (ACS) (24% vs 50%, P = 0.001), or to have more than one stent inserted into the same vessel (14% vs 19%, P = 0.002). FFR-guided PCI was significantly more likely to be performed in a private hospital compared to a public hospital (61% vs 39%, P<0.001). The baseline characteristics of the study groups are summarized in Table 1. There was no significant correlation between annual hospital PCI volume and use of FFR-guided PCI (r = -0.18, P = 0.47).

**Table 1:** Baseline characteristics of the study cohort.

	Total cohort	FFR-guided	Angio-guided	
**Parameters**	**N = 10304**	**N = 542**	**N = 9762**	**P value**
Age, years	67±12	68±11	67±12	0.60
Gender, female	2731 (27)	139 (26)	2592 (27)	0.64
**Presentation**				
Acute coronary syndrome	5033 (49)	128 (24)	4905 (50)	0.001
**Co-morbid conditions**				
Prior myocardial infarction	498 (5)	20 (4)	478 (5)	0.20
Prior PCI / CABG	736 (7)	49 (9)	687 (7)	0.08
Congestive cardiac failure	529 (5)	25 (5)	504 (5)	0.57
Stroke	51 (1)	1(0)	50 (1)	0.29
Peripheral vascular disease	258 (3)	17 (3)	241 (3)	0.33
Atrial fibrillation/flutter	616 (6)	30 (6)	586 (6)	0.66
Diabetes	2640 (26)	138 (26)	2502 (26)	0.93
Smoker, current or former	4353 (42)	226 (42)	4127 (42)	0.79
Malignancy	43 (0)	1 (0)	42 (0)	0.39
Chronic pulmonary disease	171 (2)	8 (2)	163 (2)	0.73
Neurodegenerative disease	21 (0)	0 (0)	21 (0)	0.28
Chronic kidney disease	353 (3)	11 (2)	342 (4)	0.07
**Procedural data**				
Single-vessel PCI	8626 (84)	440 (81)	8186 (84)	0.10
Multi-vessel PCI	1678 (16)	102 (19)	1576 (16)	
>1 stent to a single vessel	1929 (19)	74 (14)	1855 (19)	0.002
**Hospital type**				
Public hospital	5798 (56)	213 (39)	5585 (57)	<0.001
Private hospital	4177 (43)	329 (61)	4177 (43)	

Angio = angiography, CABG = coronary artery bypass grafting, FFR = fractional flow reserve, N = number of patients, Neurodegenerative disease = dementia, central nervous systemic atrophies, Parkinson’s disease, basal ganglia degeneration, and/or nervous systemic degenerative diseases, PCI = percutaneous coronary intervention

The baseline characteristics of the two groups were compared using the t-test for continuous variables, and Pearson’s chi square test for dichotomous variables.

### Clinical outcomes of FFR-guided vs angiography-guided PCI

**Fig 1:**
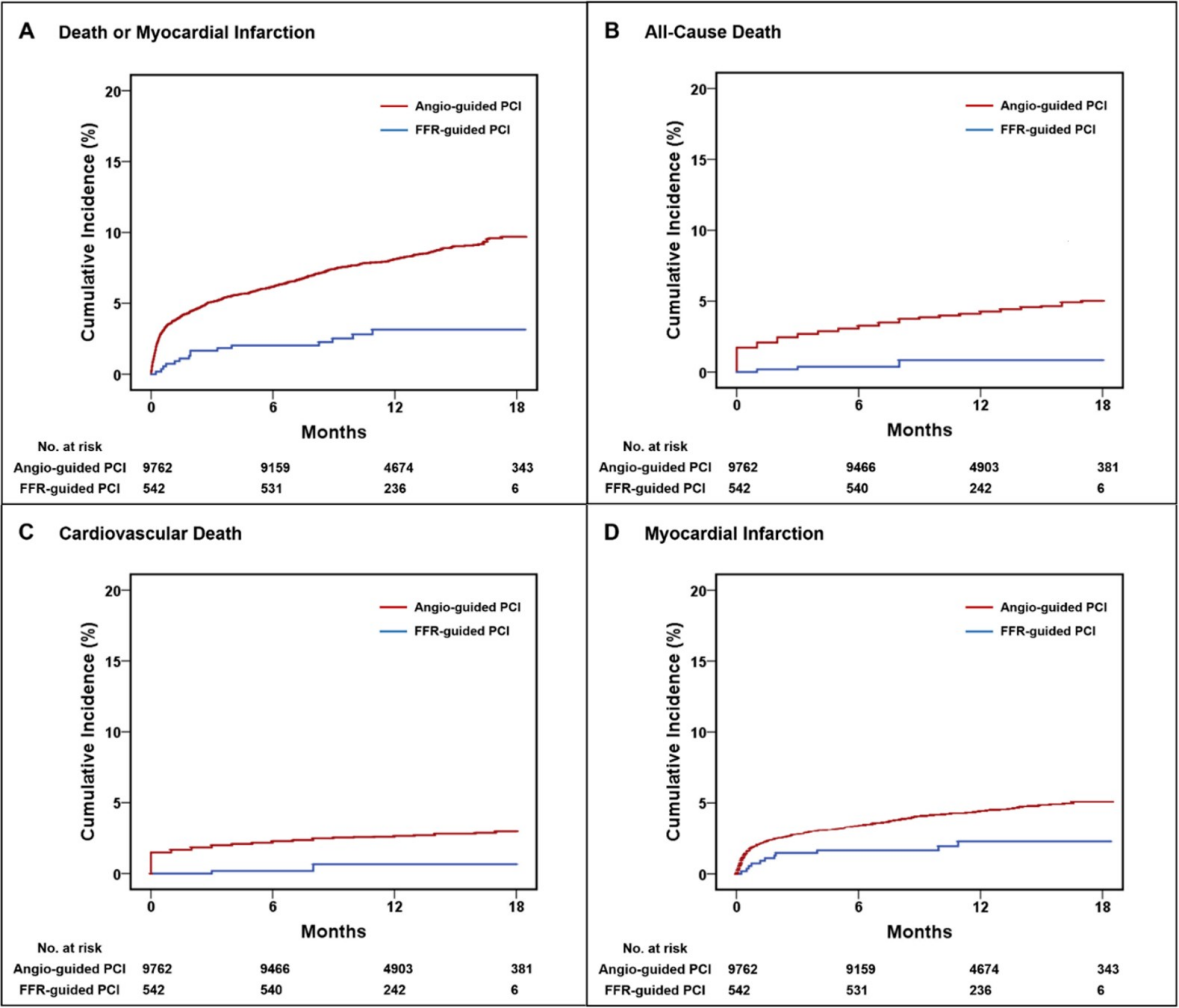
Outcomes after FFR-guided PCI compared to angiography-guided PCI. Kaplan-Meier analysis demonstrated significantly reduced occurrence of the composite endpoint of death or MI (HR 0.34, 95% CI 0.20–0.56, P<0.001) (**A**), all-cause death (HR 0.18, 95% CI 0.07–0.47, P = 0.001) (**B**), CVS death (HR 0.21, 95% CI 0.07–0.66, P = 0.01) (**C**), and MI (HR 0.46, 95% CI 0.25–0.84, P = 0.01) (**D**) in patients undergoing FFR-guided PCI vs angiography-guided PCI. Abbreviations: CVS = cardiovascular, FFR = fractional flow reserve, MI = myocardial infarction, PCI = percutaneous coronary intervention.

The mean follow-up time of the study cohort was 12±4 months. The primary outcome of all-cause death or MI occurred significantly less often in the FFR-guided group compared to the angiography-guided group (hazard ratio [HR] 0.34, 95% confidence intervals [CI] 0.20–0.56, P<0.001) (Fig 1A). Patients in the FFR-guided group also had significantly less occurrence of the secondary outcomes of all-cause death (HR 0.18, 95% CI 0.07–0.47, P = 0.001), CVS death (HR 0.21, 95% CI 0.07–0.66, P = 0.01), and MI (HR 0.46, 95% CI 0.25–0.84, P = 0.01) compared to the angiography-guided group (Fig 1B–1D). Univariable predictors of the primary and secondary outcomes are presented in S2–S5 Tables. Multivariable Cox regression adjusted analyses demonstrated that FFR-guidance was independently associated with significantly lower risk of the primary outcome (HR 0.45, 95% CI 0.27–0.75, P = 0.002) and the secondary outcomes of all-cause death (HR 0.22, 95% CI 0.08–0.59, P = 0.003) and CVS death (HR 0.27, 95% CI 0.09–0.83, P = 0.02) (Table 2). There was a trend towards lower risk of MI (HR 0.67, 95% CI 0.37–1.23, P = 0.20) associated with FFR-guidance which did not reach statistical significance. Competing risk analysis demonstrated similar sub-distribution HRs (sHR) for CVS death (sHR 0.27, Standard error = 0.57, P = 0.02) and MI (sHR 0.69, Standard error = 0.31, P = 0.23). Full details of the multivariable analyses are presented in S6–S9 Tables. There was no significant interaction between FFR-guidance and clinical presentation with stable angina or ACS (p = 0.27). Baseline characteristics and outcomes in the subgroup of patients with stable angina (S10 Table and S2 Fig) and ACS (S11 Table and S3 Fig) are presented in the Supporting Information.

**Table 2:** FFR-guidance and outcomes after PCI.

	FFR-guided	Angio-guided	Univariable	P value	Multivariable	P value
	(N = 542)	(N = 9762)	HR, 95% CI		HR, 95% CI *	
**Primary outcome**						
All-cause death or MI	15 (3%)	801 (8%)	0.34, 0.20–0.56	<0.001	0.45, 0.27–0.75	0.002
**Secondary outcomes**						
All-cause death	4 (1%)	411 (4%)	0.18, 0.07–0.47	0.001	0.22, 0.08–0.59	0.003
CVS death	3 (1%)	258 (3%)	0.21, 0.07–0.66	0.01	0.27, 0.09–0.83	0.02
MI	11 (2%)	423 (4%)	0.46, 0.25–0.84	0.01	0.67, 0.37–1.23	0.20

Angio = angiography, CI = confidence interval, CVS = cardiovascular, FFR = fractional flow reserve, HR = hazard ratio, MI = myocardial infarction, N = number of patients.

* Cox proportional hazards regression analysis was used to create multivariable models to determine whether FFR-guidance was an independent predictor of outcomes, after adjustment for all variables listed in Table 1.

The baseline characteristics of the FFR-guided (n = 542) and angiography-guided (n = 542) PCI groups after propensity score matching are presented in S12 Table. The two groups were well matched with no significant differences in demographics, clinical presentation, comorbidities, or procedural data. Patients in the FFR-guided group had significantly reduced occurrence of the primary outcome of death or MI (HR 0.32, 95% CI 0.18–0.56, P<0.001), as well as the secondary outcome of all-cause death (HR 0.11, 95% CI 0.04–0.30, P<0.001), and CVS death (HR 0.13, 95% CI 0.04–0.43, P<0.001) (S4A–S4C Fig). There was no significant difference between groups in the incidence of MI (HR 0.69, 95% CI 0.32–1.49, P = 0.55) (S4D Fig).

## Discussion

The present study examined outcomes associated with FFR-guidance in patients undergoing PCI from a large, unselected real-world cohort. The main finding was that FFR use during PCI was independently associated with a reduction in the composite endpoint of death or MI, as well as the individual endpoints of all-cause death and CVS death. The observed benefits of FFR use mainly pertain to patients with stable ischemic heart disease, although our exploratory analysis of the ACS cohort revealed trends towards reduced all-cause and CVS death as well.

Since its inception in the 1990s, FFR has gradually become the gold standard in catheter laboratories to detect myocardial ischaemia and guide revascularization decisions in stable ischemic heart disease. The current American College of Cardiology/American Heart Association guidelines give a class IIa recommendation for the use of FFR to assess the functional significance and guide revascularization in intermediate coronary stenoses [10]. However, real-world PCI practices in stable ischemic heart disease have not necessarily followed these recommendations. The use of FFR-guided PCI has steadily increased in the United States, from 8% in 2010 to 31% in 2014 [4]. In contrast, FFR guidance has not been as widely adopted in other countries, with a German registry demonstrating only 3% use in 40,160 elective PCI cases [5]. In our study, FFR was only used in 5% and 8% in the overall and stable ischemic heart disease cohort respectively, reflecting the relatively low utilization of FFR-guided PCI in Australia.

Our results corroborated findings from previous studies that demonstrated the superiority of FFR-guided PCI compared to angiography-guided PCI. The FAME study randomized 1005 patients to a routine angiography-guided PCI strategy vs an FFR-guided PCI strategy, and found a significant reduction in the composite endpoint of death or MI in the latter group, although differences in the individual endpoints of death and MI were not significant due to limited power [3]. Our stable ischemic heart disease cohort comprised 5,271 patients, and FFR-guided PCI was associated with a significant reduction in the composite of death or MI, and death alone in adjusted analyses.

Our study’s finding of a significant reduction in all-cause and CVS death can be viewed in the context of other real-world studies with large cohorts. Li et al demonstrated the use of FFR-guided intervention was associated with a 20% reduction in death or MI at a median follow-up of 4 years [11]. Fröhlich et al did not find a significant difference in mortality between patients undergoing FFR-guided PCI vs angiography-guided PCI [12]. Parikh et al showed that an FFR-guided revascularization strategy was associated with 43% reduction in 1-year mortality compared to an angiography-guided strategy in patients with intermediate stenosis, although only a minority of patients underwent revascularization [13]. Völz et al evaulated 5-year outcomes of FFR-guided versus angiography-guided PCI in patients with stable angina, and demonstrated 19% reduction in mortality at long-term follow-up in the FFR group [14]. Our results do demonstrate the largest relative reduction in mortality compared to previous studies, but differences across these real-world studies exist due to a number of factors including: 1) significant variations in PCI practice and FFR use across different geographic regions; 2) heterogeneous study design, in which some studies only included patients undergoing revascularization while others included “deferred” patients; 3) residual confounding by unaccounted differences between the FFR and angiography-guided groups. Our finding of a significant reduction in CVS death with FFR-guidance complements the current literature, as previous studies did not distinguish between CVS and non-CVS death. Despite differences in methodology and results, an overarching conclusion can be drawn that FFR use appears to be associated with reduction in mortality in contemporary real-world PCI practice.

The use of FFR in patients presenting with ACS remains a contentious issue. The main concern relates to the use of FFR in culprit vessels, as transient and reversible increases in microvascular resistance may reduce hyperemic myocardial flow and produce false negative results [15]. As a result, the choice between FFR-guided and angiography-guided PCI is not clear-cut in ACS patients. The FAMOUS-NSTEMI study evaluated 350 NSTEMI patients and measured FFR in all vessels with ≥ 30% stenosis; the cohort was then randomized to operator disclosure or blinding of the result. Patients in the FFR disclosure group had a significantly lower rate of revascularization compared to the blinded group, with no difference in major adverse cardiac events [16]. The DANAMI-3-PRIMULTI and the Compare-Acute trials both demonstrated reduced rates of major adverse cardiac events when FFR-guided PCI rather than medical therapy was used to treat non-culprit stenoses in STEMI [17, 18]. The results from the FLOWER-MI (Multivessel PCI Guided by FFR or Angiography for Myocardial Infarction) study did not show a significant benefit of FFR-guidance over angiographic-guidance in treating non-culprit lesions [19]. Results from the ongoing FRAME-AMI (FFR Versus Angiography-Guided Strategy for Management of AMI With Multivessel Disease) are eagerly awaited. In a national registry examining in-hospital mortality in patients with ACS, FFR-guidance was associated with significantly lower in-hospital mortality, although the results were likely influenced by significant confounders [20]. In our study, FFR-guidance was associated with a trend towards reduced death in patients with acute MI. However, only a small proportion of patients (3%) underwent FFR-guided PCI, and we lacked information on whether FFR was used to interrogate the culprit/non-culprit lesion and how the results affected management. Therefore, our results can only be considered exploratory and hypothesis generating, and further research into the use of FFR-guided PCI in ACS patients is warranted.

### Study limitations

Our study has several limitations. First, although we have adjusted for all possible potential confounders including patient demographics and comorbidities within the limitations of the dataset available, the retrospective design of this study raises the possibility of residual confounders that have not been accounted for. Second, our database lacked the granularity to determine the location, severity, and complexity of the treated coronary lesions, and how FFR measurement impacted on PCI decision-making. Third, FFR only began being coded into the ADPC registry from 2017 onwards, thus we were unable to examine temporal trends of FFR usage and whether outcomes changed over time. Fourth, our registry did not contain data on medication use and we were unable to adjust for differences in medical therapy between the FFR and angiography group, although differences in antiplatelet therapy would likely be minimal as all patients underwent PCI. Fifth, we were unable to adjust for individual operator experience and case volume, which could potentially account for the improved outcomes seen in the FFR-guided PCI group. It is possible that operators who perform FFR are also more likely to optimize the PCI procedure with intracoronary imaging, and therefore obtain superior results. However, the benefits of FFR-guided PCI remained significant after adjusting for different hospital sites, and we also did not observe a significant correlation between hospital PCI volume and the use of FFR-guided PCI. Sixth, our study lacked a clinical events committee. Finally, it is surprising that FFR-guided PCI was associated with a significant reduction in all-cause and CVS death, with only a trend towards less MI. This lack of difference in MI rates was also observed in the study by Parikh et al [13], and we believe this discrepancy may be attributed to the following reasons: Patients that died in the community from sudden CVS causes before receiving medical attention may lead to underestimation of the true MI rate; real-world PCI operators may have a subconscious bias in selecting patients with less comorbidities and complex lesions for FFR-guidance, which could have influenced outcomes despite our best attempts at adjustment. Nevertheless, our finding of a significant reduction in CVS-death provides some reassurance that the reduction in mortality seen in the FFR group may reflect the benefits from the procedure itself rather than selection bias.

## Conclusions

In this real-world study on FFR vs angiography-guided PCI in an unselected population, FFR-guidance was independently associated with a significant reduction in the combined endpoint of death or MI, as well as a reduction in the individual endpoints of all-cause death and CVS death. Our results are consistent with previous studies and provide further impetus for increasing the adoption of FFR-guided PCI in clinical practice.

## Supporting information

S1 Fig Study design flowchart. Abbreviations: FFR = fractional flow reserve, N = number, NSW = New South Wales, PCI = percutaneous coronary intervention.
(DOCX)

S2 Fig Outcomes after FFR-guided PCI compared to angiography-guided PCI in patients with stable ischemic heart disease. Kaplan-Meier analysis demonstrated significantly reduced occurrence of the composite endpoint of death or MI (HR 0.29, P = 0.002) **(A)**, all-cause death (HR 0.15, P = 0.002) **(B)**, and CVS death (HR 0.27, P = 0.046) **(C)** in patients undergoing FFR-guided PCI vs angiography-guided PCI. There was no significant difference in MI (HR 0.50, P = 0.17) between the two groups **(D)**. Abbreviations: CVS = cardiovascular, FFR = fractional flow reserve, MI = myocardial infarction, PCI = percutaneous coronary intervention.
(DOCX)

S3 Fig Outcomes after FFR-guided PCI compared to angiography-guided PCI in patients with acute coronary syndrome. Kaplan-Meier survival analysis with the log rank test demonstrated no significant differences between the FFR-guided PCI and angiography-guided PCI groups with respect to the composite endpoint of death or MI (HR 0.61, P = 0.13) **(A)**, all-cause death (HR 0.30, P = 0.07), CVS death (HR 0.23, P = 0.10) **(C)**, or MI (HR 0.80, P = 0.56) **(D)**. Abbreviations: CVS = cardiovascular, FFR = fractional flow reserve, MI = myocardial infarction, PCI = percutaneous coronary intervention.
(DOCX)

S4 Fig Kaplan Meier survival curves of the propensity score-matched FFR- and angiography-guided PCI groups. Kaplan-Meier survival analysis demonstrating significant reduction in the primary endpoint of death or MI (HR 0.32, P<0.001) **(A)**, all-cause death (HR 0.11, P<0.001) **(B)**, and CVS death (HR 0.13, P<0.001) **(C)** associated with the FFR-guided PCI group. There was no significant difference in MI between the FFR-guided PCI and angiography-guided PCI group (HR 0.69, P = 0.55) **(D)**. Abbreviations: CVS = cardiovascular, FFR = fractional flow reserve, MI = myocardial infarction, PCI = percutaneous coronary intervention.
(DOCX)

S1 Table Centre-for-Health-Record-Linkage Population-Linkage Study Morbidities; defined by International Classification of Diseases Tenth Revision Australian Modification Codes & Australian Classification of Health Interventions Procedural Codes. (DOCX)

S2 Table Univariable predictors of the primary outcome. AF = atrial fibrillation, CABG = coronary artery bypass grafting, CI = confidence interval, FFR = fractional flow reserve, HR = hazard ratio, Neurodegenerative disease = dementia, central nervous systemic atrophies, Parkinson’s disease, basal ganglia degeneration, and/or nervous systemic degenerative diseases, PCI = percutaneous coronary intervention. Cox proportional hazards regression analysis was used to determine the hazard ratio of individual variables.
(DOCX)

S3 Table Univariable predictors of all-cause death. AF = atrial fibrillation, CABG = coronary artery bypass grafting, CI = confidence interval, FFR = fractional flow reserve, HR = hazard ratio, Neurodegenerative disease = dementia, central nervous systemic atrophies, Parkinson’s disease, basal ganglia degeneration, and/or nervous systemic degenerative diseases, PCI = percutaneous coronary intervention. Cox proportional hazards regression analysis was used to determine the hazard ratio of individual variables.
(DOCX)

S4 Table Univariable predictors of CVS death. AF = atrial fibrillation, CABG = coronary artery bypass grafting, CI = confidence interval, FFR = fractional flow reserve, HR = hazard ratio, Neurodegenerative disease = dementia, central nervous systemic atrophies, Parkinson’s disease, basal ganglia degeneration, and/or nervous systemic degenerative diseases, PCI = percutaneous coronary intervention. Cox proportional hazards regression analysis was used to determine the hazard ratio of individual variables.
(DOCX)

S5 Table Univariable predictors of MI. AF = atrial fibrillation, CABG = coronary artery bypass grafting, CI = confidence interval, FFR = fractional flow reserve, HR = hazard ratio, Neurodegenerative disease = dementia, central nervous systemic atrophies, Parkinson’s disease, basal ganglia degeneration, and/or nervous systemic degenerative diseases, PCI = percutaneous coronary intervention. Cox proportional hazards regression analysis was used to determine the hazard ratio of individual variables.
(DOCX)

S6 Table Multivariable predictors of the primary outcome. AF = atrial fibrillation, CABG = coronary artery bypass grafting, CI = confidence interval, FFR = fractional flow reserve, HR = hazard ratio, Neurodegenerative disease = dementia, central nervous systemic atrophies, Parkinson’s disease, basal ganglia degeneration, and/or nervous systemic degenerative diseases, PCI = percutaneous coronary intervention. Cox proportional hazards regression analysis was used to determine the hazard ratio of individual variables.
(DOCX)

S7 Table Multivariable predictors of all-cause death. AF = atrial fibrillation, CABG = coronary artery bypass grafting, CI = confidence interval, FFR = fractional flow reserve, HR = hazard ratio, Neurodegenerative disease = dementia, central nervous systemic atrophies, Parkinson’s disease, basal ganglia degeneration, and/or nervous systemic degenerative diseases, PCI = percutaneous coronary intervention. Cox proportional hazards regression analysis was used to determine the hazard ratio of individual variables.
(DOCX)

S8 Table Multivariable predictors of CVS death. AF = atrial fibrillation, CABG = coronary artery bypass grafting, CI = confidence interval, FFR = fractional flow reserve, HR = hazard ratio, Neurodegenerative disease = dementia, central nervous systemic atrophies, Parkinson’s disease, basal ganglia degeneration, and/or nervous systemic degenerative diseases, PCI = percutaneous coronary intervention. Cox proportional hazards regression analysis was used to determine the hazard ratio of individual variables.
(DOCX)

S9 Table Multivariable predictors of MI. AF = atrial fibrillation, CABG = coronary artery bypass grafting, CI = confidence interval, FFR = fractional flow reserve, HR = hazard ratio, Neurodegenerative disease = dementia, central nervous systemic atrophies, Parkinson’s disease, basal ganglia degeneration, and/or nervous systemic degenerative diseases, PCI = percutaneous coronary intervention. Cox proportional hazards regression analysis was used to determine the hazard ratio of individual variables.
(DOCX)

S10 Table Baseline characteristics of the stable ischemic heart disease cohort. Angio = angiography, CABG = coronary artery bypass grafting, FFR = fractional flow reserve, N = number of patients, Neurodegenerative disease = dementia, central nervous systemic atrophies, Parkinson’s disease, basal ganglia degeneration, and/or nervous systemic degenerative diseases, PCI = percutaneous coronary intervention. The baseline characteristics of the two groups were compared using the t-test for continuous variables, and Pearson’s chi square test for dichotomous variables.
(DOCX)

S11 Table Baseline characteristics of the acute coronary syndrome cohort. Angio = angiography, CABG = coronary artery bypass grafting, FFR = fractional flow reserve, N = number of patients, Neurodegenerative disease = dementia, central nervous systemic atrophies, Parkinson’s disease, basal ganglia degeneration, and/or nervous systemic degenerative diseases, PCI = percutaneous coronary intervention. The baseline characteristics of the two groups were compared using the t-test for continuous variables, and Pearson’s chi square test for dichotomous variables.
(DOCX)

S12 Table Baseline characteristics of the propensity-matched cohort. Angio = angiography, CABG = coronary artery bypass grafting, FFR = fractional flow reserve, N = number of patients, Neurodegenerative disease = dementia, central nervous systemic atrophies, Parkinson’s disease, basal ganglia degeneration, and/or nervous systemic degenerative diseases, PCI = percutaneous coronary intervention. The baseline characteristics of the two groups were compared using the t-test for continuous variables, and Pearson’s chi square test for dichotomous variables.
(DOCX)

## Abbreviations list

ACS: acute coronary syndrome
APDC: Admission Patient Data Collection
CI: confidence interval
CVS: cardiovascular
FFR: fractional flow reserve
HR: hazard ratio
MI: myocardial infarction
NSW: New South Wales
PCI: percutaneous coronary intervention
sHR: sub-distribution hazard ratio
