# Supplementary material for: A real-world comparison of outcomes between fractional flow reserve-guided versus angiography-guided percutaneous coronary intervention
Source: PLoS One. 2021 Dec 16;16(12):e0259662. doi: 10.1371/journal.pone.0259662 (PMC8675732; doi:10.1371/journal.pone.0259662)
Supplement: S1 Fig — Abbreviations: FFR = fractional flow reserve, N = number, NSW = New South Wales, PCI = percutaneous coronary intervention. (DOCX) [file pone.0259662.s001.docx]

**S1 Fig:** Study design flowchart

**
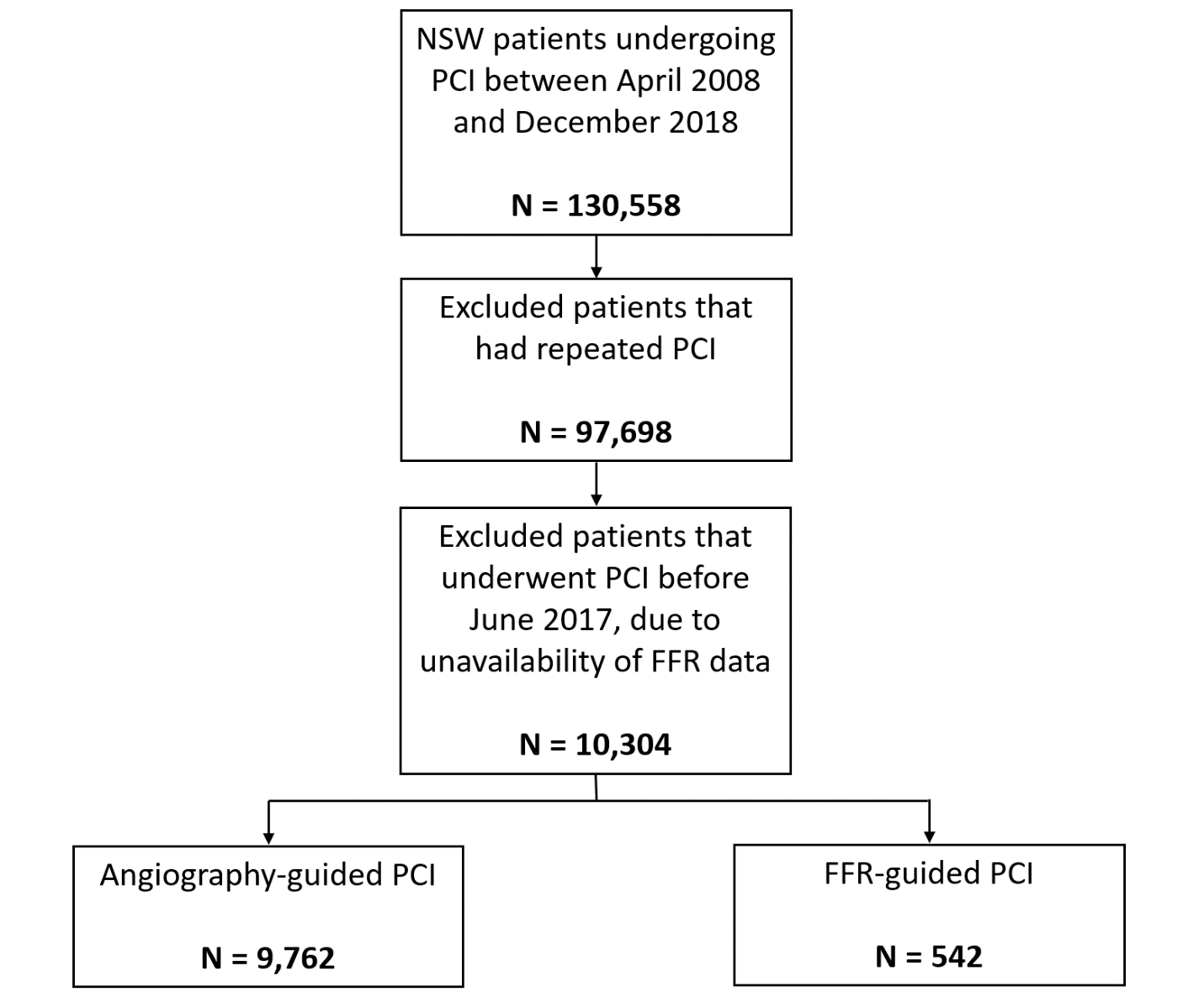
**

Abbreviations: FFR = fractional flow reserve, N = number, NSW = New South Wales, PCI = percutaneous coronary intervention,
