# Supplementary material for: A real-world comparison of outcomes between fractional flow reserve-guided versus angiography-guided percutaneous coronary intervention
Source: PLoS One. 2021 Dec 16;16(12):e0259662. doi: 10.1371/journal.pone.0259662 (PMC8675732; doi:10.1371/journal.pone.0259662)
Supplement: S2 Fig — Kaplan-Meier analysis demonstrated significantly reduced occurrence of the composite endpoint of death or MI (HR 0.29, P = 0.002) (A), all-cause death (HR 0.15, P = 0.002) (B), and CVS death (HR 0.27, P = 0.046) (C) in patients undergoing FFR-guided PCI vs angiography-guided PCI. There was no significant difference in MI (HR 0.50, P = 0.17) between the two groups (D). Abbreviations: CVS = cardiovascular, FFR = fractional flow reserve, MI = myocardial infarction, PCI = percutaneous coronary intervention. (DOCX) [file pone.0259662.s002.docx]

**S2 Fig:** Outcomes after FFR-guided PCI compared to angiography-guided PCI in patients with stable ischemic heart disease.

**
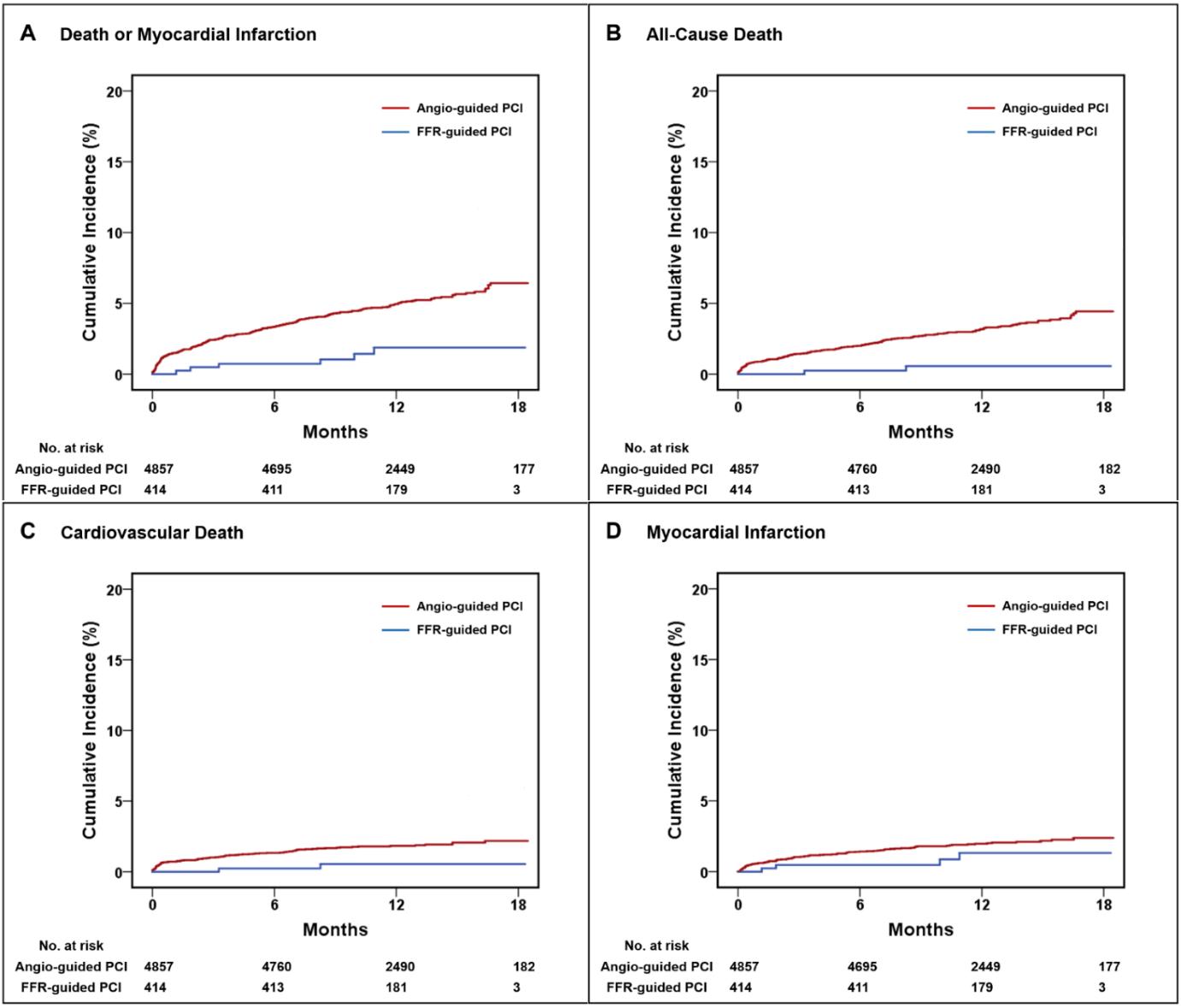
**

Caption: Kaplan-Meier analysis demonstrated significantly reduced occurrence of the composite endpoint of death or MI (HR 0.29, P=0.002) **(A)**, all-cause death (HR 0.15, P=0.002) **(B)**, and CVS death (HR 0.27, P=0.046) **(C)** in patients undergoing FFR-guided PCI vs angiography-guided PCI. There was no significant difference in MI (HR 0.50, P=0.17) between the two groups **(D)**.

Abbreviations: CVS = cardiovascular, FFR = fractional flow reserve, MI = myocardial infarction, PCI = percutaneous coronary intervention
