# Supplementary material for: A real-world comparison of outcomes between fractional flow reserve-guided versus angiography-guided percutaneous coronary intervention
Source: PLoS One. 2021 Dec 16;16(12):e0259662. doi: 10.1371/journal.pone.0259662 (PMC8675732; doi:10.1371/journal.pone.0259662)
Supplement: S3 Fig — Kaplan-Meier survival analysis with the log rank test demonstrated no significant differences between the FFR-guided PCI and angiography-guided PCI groups with respect to the composite endpoint of death or MI (HR 0.61, P = 0.13) (A), all-cause death (HR 0.30, P = 0.07), CVS death (HR 0.23, P = 0.10) (C), or MI (HR 0.80, P = 0.56) (D). Abbreviations: CVS = cardiovascular, FFR = fractional flow reserve, MI = myocardial infarction, PCI = percutaneous coronary intervention. (DOCX) [file pone.0259662.s003.docx]

**S3 Fig:** Outcomes after FFR-guided PCI compared to angiography-guided PCI in patients with acute coronary syndrome.


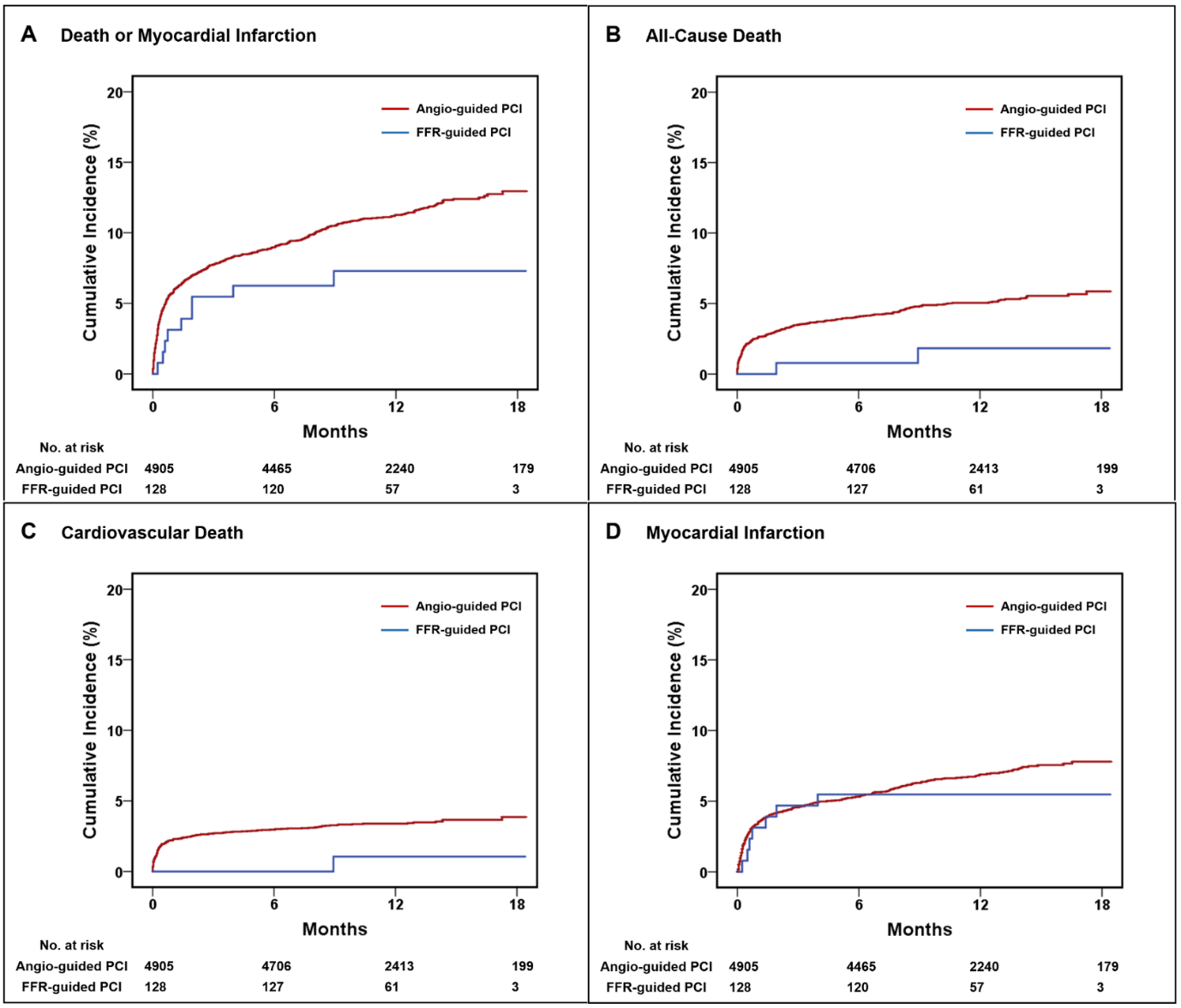


Caption: Kaplan-Meier survival analysis with the log rank test demonstrated no significant differences between the FFR-guided PCI and angiography-guided PCI groups with respect to the composite endpoint of death or MI (HR 0.61, P=0.13) **(A)**, all-cause death (HR 0.30, P=0.07), CVS death (HR 0.23, P=0.10) **(C)**, or MI (HR 0.80, P=0.56) **(D)**.

Abbreviations: CVS = cardiovascular, FFR = fractional flow reserve, MI = myocardial infarction, PCI = percutaneous coronary intervention
