# Supplementary material for: A real-world comparison of outcomes between fractional flow reserve-guided versus angiography-guided percutaneous coronary intervention
Source: PLoS One. 2021 Dec 16;16(12):e0259662. doi: 10.1371/journal.pone.0259662 (PMC8675732; doi:10.1371/journal.pone.0259662)
Supplement: S4 Fig — Kaplan-Meier survival analysis demonstrating significant reduction in the primary endpoint of death or MI (HR 0.32, P<0.001) (A), all-cause death (HR 0.11, P<0.001) (B), and CVS death (HR 0.13, P<0.001) (C) associated with the FFR-guided PCI group. There was no significant difference in MI between the FFR-guided PCI and angiography-guided PCI group (HR 0.69, P = 0.55) (D). Abbreviations: CVS = cardiovascular, FFR = fractional flow reserve, MI = myocardial infarction, PCI = percutaneous coronary intervention. (DOCX) [file pone.0259662.s004.docx]

**S4 Fig:** Kaplan Meier survival curves of the propensity score-matched FFR- and angiography-guided PCI groups

*
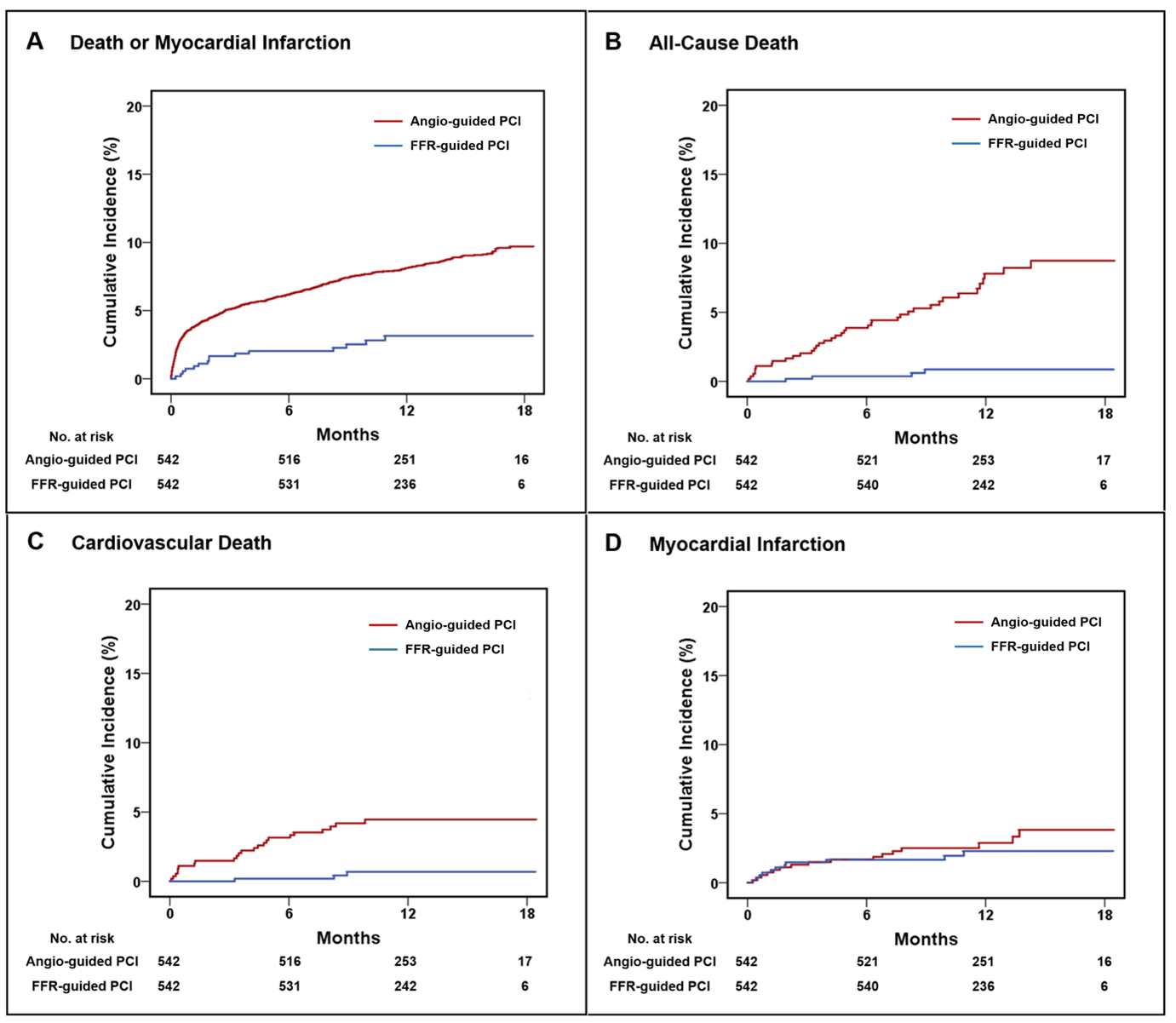
*

Caption: Kaplan-Meier survival analysis demonstrating significant reduction in the primary endpoint of death or MI (HR 0.32, P<0.001) **(A)**, all-cause death (HR 0.11, P<0.001) **(B)**, and CVS death (HR 0.13, P<0.001) **(C)** associated with the FFR-guided PCI group. There was no significant difference in MI between the FFR-guided PCI and angiography-guided PCI group (HR 0.69, P=0.55) **(D)**. Abbreviations: CVS = cardiovascular, FFR = fractional flow reserve, MI = myocardial infarction, PCI = percutaneous coronary intervention
