# Supplementary material for: A real-world comparison of outcomes between fractional flow reserve-guided versus angiography-guided percutaneous coronary intervention
Source: PLoS One. 2021 Dec 16;16(12):e0259662. doi: 10.1371/journal.pone.0259662 (PMC8675732; doi:10.1371/journal.pone.0259662)
Supplement: S1 Table — (DOCX) [file pone.0259662.s005.docx]

**S1 Table:** Centre-for-Health-Record-Linkage Population-Linkage Study Morbidities; defined by International Classification of Diseases Tenth Revision Australian Modification Codes & Australian Classification of Health Interventions Procedural Codes

| **No.** | **Morbidity** | **International Classification of Diseases Tenth Revision Australian Modification** |
| --- | --- | --- |
| 1 | Atrial fibrillation and/or flutter | I48, I48.0, I48.1, I48.2, I48.3, I48.4, I48.9 |
| 2 | Acute coronary syndrome | I20.0, I21, I21.0, I21.1, I21.2, I21.3, I21.4, I21.9, I22, I22.0, I22.1, I22.8, I22.9 |
| 3 | Prior/presence of coronary artery bypass graft | Z95.1 |
| 4 | Prior/presence of coronary angioplasty implant and graft | Z95.5 |
| 5 | Prior/presence of coronary artery bypass graft and/or coronary angioplasty implant and graft | Z95.1, Z95.5 |
| 6 | Congestive cardiac failure | I42, I42.0, I42.1, I42.2, I42.3, I42.4, I42.5, I42.6, I42.7, I42.8, I42.9, I43, I43.0, I43.1, I43.2, I43.8, I50, I50.0, I50.1, I50.9, I11.0, I13.0, I13.2 |
| 7 | Peripheral vascular disease | E09.5, E09.51, E09.52, E10.51, E10.52, E11.51, E11.52, E13.51, E13.52, E14.51, E14.52, I70, I70.0, I70.1, I70.2, I70.20, I70.21, I70.22, I70.23, I70.24, I70.8, I70.9, I71, I71.0, I71.00, I71.01, I71.02, I71.03, I71.1, I71.2, I71.3, I71.4, I71.5, I71.6, I71.8, I71.9, I72, I72.0 I72.1, I72.2, I72.3, I72.4, I72.5, I72.6, I72.8, I72.9, I73, I73.0, I73.1, I73.8, I73.9, I74, I74.0, I74.1, I74.2, I74.3, I74.4, I74.5, I74.8, I74.9, I77, I77.0, I77.1, I77.2, I77.3, I77.4, I77.5, I77.6, I77.8, I77.9, I78, I78.0, I78.1, I78.8, I78.9, I79, I79.0, I79.1, I79.2, I79.8 |
| 8 | Cerebrovascular accident/disease | G45, G45.0, G45.1, G45.2, G45.3, G45.4, G45.8, G45.9, G46, G46.0, G46.1, G46.2, G46.3, G46.4, G46.5, G46.6, G46.7, G46.8, I60, I60.0, I60.1, I60.2, I60.3, I60.4, I60.5, I60.6, I60.7, I60.8, I60.9, I61, I61.0, I61.1, I61.2, I61.3, I61.4, I61.5, I61.6, I61.8, I61.9, I62, I62.0, I62.1, I62.9, I63, I63.0, I63.1, I63.2, I63.3, I63.4, I63.5, I63.6, I63.8, I63.9, I64 |
| 9 | Diabetes | E09, E09.2, E09.21, E09.29, E09.3, E09.31, E09.32, E09.4, E09.40, E09.42, E09.5, E09.51, E09.52, E09.7, E09.71, E09.72, E09.8, E09.9, E10, E10.0, E10.01, E10.02, E10.1, E10.11, E10.12, E10.13, E10.14, E10.15, E10.16, E10.2, E10.21, E10.22, E10.29, E10.3, E10.31, E10.32, E10.33, E10.34, E10.35, E10.36, E10.39, E10.4, E10.40, E10.41, E10.42, E10.43, E10.49, E10.5, E10.51, E10.52, E10.53, E10.6, E10.61, E10.62, E10.63, E10.64, E10.65, E10.69, E10.7, E10.71, E10.73, E10.8, E10.9, E11, E11.0, E11.01, E11.02, E11.1, E11.11, E11.12, E11.13, E11.14, E11.15, E11.16, E11.2, E11.21, E11.22, E11.29, E11.3, E11.31, E11.32, E11.33, E11.34, E11.35, E11.36, E11.39, E11.4, E11.40, E11.41, E11.42, E11.43, E11.49, E11.5, E11.51, E11.52, E11.53, E11.6, E11.61, E11.62, E11.63, E11.64, E11.65, E11.69, E11.7, E11.71, E11.72, E11.73, E11.8, E11.9, E13, E13.0, E13.01, E13.02, E13.1, E13.11, E13.12, E13.13, E13.14, E13.15, E13.16, E13.2, E13.21, E13.22, E13.29, E13.3, E13.31, E13.32, E13.33, E13.34, E13.35, E13.36, E13.39, E13.4, E13.40, E13.41, E13.42, E13.43, E13.49, E13.5, E13.51, E13.52, E13.53, E13.6, E13.61, E13.62, E13.63, E13.64, E13.65, E13.69, E13.7, E13.71, E13.72, E13.73, E13.8, E13.9, E14, E14.0, E14.01, E14.02, E14.1, E14.11, E14.12, E14.13, E14.14, E14.15, E14.16, E14.2, E14.21, E14.22, E14.29, E14.3, E14.31, E14.32, E14.33, E14.34, E14.35, E14.36, E14.39, E14.4, E14.40, E14.41, E14.42, E14.43, E14.49, E14.5, E14.51, E14.52, E14.53, E14.6, E14.61, E14.62, E14.63, E14.64, E14.65, E14.69, E14.7, E14.71, E14.72, E14.73, E14.8, E14.9, Z92.22 |
| 10 | Current and/or ex-smoker | F17, Z72.0, Z86.43 |
| 11 | Chronic pulmonary disease (includes asthma, chronic airways limitation, interstitial lung disease, cystic fibrosis with pulmonary manifestation) | E84.0, E84.0, E84.1, E84.8, E84.9, J40, J41, J41.0, J41.1, J41.8, J42, J43, J43.0, J43.1, J43.2, J43.8, J43.9, J44, J44.0, J44.1, J44.8, J44.9, J45, J45.0, J45.1, J45.8, J45.9, J46, J47, J60, J61, J62, J62.0, J62.8, J63, J63.0, J63.1, J63.2, J63.3, J63.4, J63.5, J63.8, J64, J65, J66, J66.0, J66.1, J66.2, J66.8, J67, J67.0, J67.1, J67.2, J67.3, J67.4, J67.5, J67.6, J67.7, J67.8, J67.9, J68, J68.0, J68.1, J68.2, J68.3, J68.4, J68.8, J68.9, J70, J70.0, J70.1, J70.2, J70.3, J70.4, J70.8, J70.9, J82, J84, J84.0, J84.1, J84.8. J84.9, J99, J99.1, J99.8 |
| 12 | Malignancy | C00-C96, D00-D09 |
| 13 | Chronic kidney disease | N18, N18.1, N18.2, N18.3, N18.4, N18.5, N18.9, N19 |
| 14 | Neurodegenerative diseases (includes dementia, central nervous systemic atrophies, Parkinson’s disease, basal ganglia degeneration and/or nervous systemic degenerative diseases) | F00, F00.0, F00.1, F00.2, F00.9, F01, F01.0, F01.1, F01.2, F01.3, F01.8, F01.9, F02, F02.0, F02.1, F02.2, F02.3, F02.4, F02.8, F03, G10, G11, G11.0, G11.1, G11.2, G11.3, G11.4, G11.8, G11.9, G12, G12.0, G12.1, G12.2, G12.8, G12.9, G13, G13.0, G13.1, G13.2, G13.8, G14, G20, G23, G23.0, G23.1, G23.2, G23.8, G23.9, G30, G30.0, G30.1, G30.8, G30.9, G31, G31.0, G31.1, G31.2, G31.3, G31.8, G31.9 |
| **No.** | **Procedures** | **Australian Classification of Health Interventions Procedural Codes** |
| 15 | Percutaneous coronary intervention  (transluminal coronary angioplasty and transluminal coronary angioplasty with stenting) | 38300-00, 38303-00, 38306-00, 38306-01, 38306-02 |
| 16 | Percutaneous coronary intervention subcategory:   - Transluminal balloon angioplasty of single coronary artery | 38300-00 |
| 17 | Percutaneous coronary intervention subcategory:   - Transluminal balloon angioplasty of more than one coronary artery | 38303-00 |
| 18 | Percutaneous coronary intervention subcategory:   - Transluminal insertion of one stent into single coronary artery | 38306-00 |
| 19 | Percutaneous coronary intervention subcategory:   - Transluminal insertion of more than one stent into single coronary artery | 38306-01 |
| 20 | Percutaneous coronary intervention subcategory:   - Transluminal insertion of more than one stent into more than one coronary artery | 38306-02 |
| 21 | Fractional flow reserve | 38241-00 |
