# Supplementary material for: A real-world comparison of outcomes between fractional flow reserve-guided versus angiography-guided percutaneous coronary intervention
Source: PLoS One. 2021 Dec 16;16(12):e0259662. doi: 10.1371/journal.pone.0259662 (PMC8675732; doi:10.1371/journal.pone.0259662)
Supplement: S2 Table — AF = atrial fibrillation, CABG = coronary artery bypass grafting, CI = confidence interval, FFR = fractional flow reserve, HR = hazard ratio, Neurodegenerative disease = dementia, central nervous systemic atrophies, Parkinson’s disease, basal ganglia degeneration, and/or nervous systemic degenerative diseases, PCI = percutaneous coronary intervention. Cox proportional hazards regression analysis was used to determine the hazard ratio of individual variables. (DOCX) [file pone.0259662.s006.docx]

**S2 Table:** Univariable predictors of the primary outcome

| **Parameters** | **HR** | **95% CI** | **P value** |
| --- | --- | --- | --- |
| Age, per-1-year increase | 1.03 | 1.03 – 1.04 | <0.001 |
| Female sex | 0.96 | 0.82 – 1.12 | 0.59 |
| **Clinical presentation** |  |  |  |
| Acute coronary syndrome | 2.48 | 2.14 – 2.88 | <0.001 |
| **Comorbidities** |  |  |  |
| Prior myocardial infarction | 1.98 | 1.56 – 2.52 | <0.001 |
| Prior CABG or PCI | 0.96 | 0.73 – 1.25 | 0.74 |
| Heart failure | 4.23 | 3.52 – 5.08 | <0.001 |
| AF/Atrial flutter | 2.67 | 2.18 – 3.26 | <0.001 |
| Stroke | 4.71 | 2.82 – 7.84 | <0.001 |
| Peripheral vascular disease | 2.34 | 1.73 – 3.17 | <0.001 |
| Diabetes | 1.29 | 1.12 – 1.50 | 0.001 |
| Smoker, current or former | 0.89 | 0.78 – 1.03 | 0.12 |
| Chronic kidney disease | 3.97 | 3.20 – 4.92 | <0.001 |
| Chronic lung disease | 3.26 | 2.37 – 4.48 | <0.001 |
| Malignancy | 6.20 | 3.89 – 9.89 | <0.001 |
| Neurodegenerative disease | 3.99 | 1.79 – 8.92 | 0.001 |
| **Procedural data** |  |  |  |
| FFR-guidance | 0.34 | 0.20 – 0.56 | <0.001 |
| Multi-vessel PCI | 1.24 | 1.04 – 1.48 | 0.02 |
| >1 stent to a single vessel | 1.13 | 0.96 – 1.34 | 0.15 |
| **Hospital type** |  |  |  |
| Private hospital | 0.59 | 0.51 – 0.69 | <0.001 |

AF = atrial fibrillation, CABG = coronary artery bypass grafting, CI = confidence interval, FFR = fractional flow reserve, HR = hazard ratio, Neurodegenerative disease = dementia, central nervous systemic atrophies, Parkinson’s disease, basal ganglia degeneration, and/or nervous systemic degenerative diseases, PCI = percutaneous coronary intervention

Cox proportional hazards regression analysis was used to determine the hazard ratio of individual variables.
