# Supplementary material for: A real-world comparison of outcomes between fractional flow reserve-guided versus angiography-guided percutaneous coronary intervention
Source: PLoS One. 2021 Dec 16;16(12):e0259662. doi: 10.1371/journal.pone.0259662 (PMC8675732; doi:10.1371/journal.pone.0259662)
Supplement: S3 Table — AF = atrial fibrillation, CABG = coronary artery bypass grafting, CI = confidence interval, FFR = fractional flow reserve, HR = hazard ratio, Neurodegenerative disease = dementia, central nervous systemic atrophies, Parkinson’s disease, basal ganglia degeneration, and/or nervous systemic degenerative diseases, PCI = percutaneous coronary intervention. Cox proportional hazards regression analysis was used to determine the hazard ratio of individual variables. (DOCX) [file pone.0259662.s007.docx]

**S3 Table:** Univariable predictors of all-cause death

| **Parameters** | **HR** | **95% CI** | **P value** |
| --- | --- | --- | --- |
| Age, per-1-year increase | 1.06 | 1.05 – 1.07 | <0.001 |
| Female sex | 0.94 | 0.76 – 1.17 | 0.59 |
| **Clinical presentation** |  |  |  |
| Acute coronary syndrome | 1.65 | 1.36 – 2.01 | <0.001 |
| **Comorbidities** |  |  |  |
| Prior myocardial infarction | 2.68 | 1.99 – 3.61 | <0.001 |
| Prior CABG or PCI | 0.94 | 0.64 – 1.39 | 0.77 |
| Heart failure | 6.74 | 5.40 – 8.42 | <0.001 |
| AF/Atrial flutter | 3.34 | 2.58 – 4.31 | <0.001 |
| Stroke | 7.90 | 4.55 – 13.73 | <0.001 |
| Peripheral vascular disease | 4.04 | 2.88 – 5.67 | <0.001 |
| Diabetes | 1.30 | 1.05 – 1.60 | 0.01 |
| Smoker, current or former | 0.87 | 0.72 – 1.06 | 0.17 |
| Chronic kidney disease | 5.18 | 3.95 – 6.79 | <0.001 |
| Chronic lung disease | 5.09 | 3.53 – 7.34 | <0.001 |
| Malignancy | 11.12 | 6.75 – 18.34 | <0.001 |
| Neurodegenerative disease | 8.01 | 3.58 – 17.93 | <0.001 |
| **Procedural data** |  |  |  |
| FFR-guidance | 0.18 | 0.07 – 0.47 | 0.001 |
| Multi-vessel PCI | 1.28 | 1.01 – 1.63 | 0.046 |
| >1 stent to a single vessel | 1.26 | 1.00 – 1.59 | 0.046 |
| **Hospital type** |  |  |  |
| Private hospital | 0.50 | 0.41 – 0.62 | <0.001 |

AF = atrial fibrillation, CABG = coronary artery bypass grafting, CI = confidence interval, FFR = fractional flow reserve, HR = hazard ratio, Neurodegenerative disease = dementia, central nervous systemic atrophies, Parkinson’s disease, basal ganglia degeneration, and/or nervous systemic degenerative diseases, PCI = percutaneous coronary intervention

Cox proportional hazards regression analysis was used to determine the hazard ratio of individual variables.
