# Supplementary material for: A real-world comparison of outcomes between fractional flow reserve-guided versus angiography-guided percutaneous coronary intervention
Source: PLoS One. 2021 Dec 16;16(12):e0259662. doi: 10.1371/journal.pone.0259662 (PMC8675732; doi:10.1371/journal.pone.0259662)
Supplement: S4 Table — AF = atrial fibrillation, CABG = coronary artery bypass grafting, CI = confidence interval, FFR = fractional flow reserve, HR = hazard ratio, Neurodegenerative disease = dementia, central nervous systemic atrophies, Parkinson’s disease, basal ganglia degeneration, and/or nervous systemic degenerative diseases, PCI = percutaneous coronary intervention. Cox proportional hazards regression analysis was used to determine the hazard ratio of individual variables. (DOCX) [file pone.0259662.s008.docx]

**S4 Table.** Univariable predictors of CVS death

| **Parameters** | **HR** | **95% CI** | **P value** |
| --- | --- | --- | --- |
| Age, per-1-year increase | 1.04 | 1.03 – 1.05 | <0.001 |
| Female sex | 0.83 | 0.64 – 1.08 | 0.17 |
| **Clinical presentation** |  |  |  |
| Acute coronary syndrome | 1.94 | 1.51 – 2.50 | <0.001 |
| **Comorbidities** |  |  |  |
| Prior myocardial infarction | 2.59 | 1.77 – 3.79 | <0.001 |
| Prior CABG or PCI | 1.02 | 0.64 – 1.63 | 0.93 |
| Heart failure | 8.86 | 6.82 – 11.52 | <0.001 |
| AF/Atrial flutter | 4.04 | 2.98 – 5.48 | <0.001 |
| Stroke | 7.28 | 3.60 – 14.71 | <0.001 |
| Peripheral vascular disease | 3.37 | 2.13 – 5.31 | <0.001 |
| Diabetes | 1.36 | 1.05 – 1.76 | 0.02 |
| Smoker, current or former | 0.73 | 0.57 – 0.94 | 0.02 |
| Chronic kidney disease | 4.57 | 3.20 – 6.52 | <0.001 |
| Chronic lung disease | 4.32 | 2.64 – 7.06 | <0.001 |
| Malignancy | 6.26 | 2.79 – 14.06 | <0.001 |
| Neurodegenerative disease | 6.13 | 1.97 – 19.15 | 0.002 |
| **Procedural data** |  |  |  |
| FFR-guidance | 0.21 | 0.07 – 0.66 | 0.01 |
| Multi-vessel PCI | 1.23 | 0.90 – 1.67 | 0.19 |
| >1 stent to a single vessel | 1.39 | 1.05 – 1.85 | 0.02 |
| **Hospital type** |  |  |  |
| Private hospital | 0.41 | 0.31 – 0.55 | <0.001 |

AF = atrial fibrillation, CABG = coronary artery bypass grafting, CI = confidence interval, FFR = fractional flow reserve, HR = hazard ratio, Neurodegenerative disease = dementia, central nervous systemic atrophies, Parkinson’s disease, basal ganglia degeneration, and/or nervous systemic degenerative diseases, PCI = percutaneous coronary intervention

Cox proportional hazards regression analysis was used to determine the hazard ratio of individual variables.
