# Supplementary material for: A real-world comparison of outcomes between fractional flow reserve-guided versus angiography-guided percutaneous coronary intervention
Source: PLoS One. 2021 Dec 16;16(12):e0259662. doi: 10.1371/journal.pone.0259662 (PMC8675732; doi:10.1371/journal.pone.0259662)
Supplement: S5 Table — AF = atrial fibrillation, CABG = coronary artery bypass grafting, CI = confidence interval, FFR = fractional flow reserve, HR = hazard ratio, Neurodegenerative disease = dementia, central nervous systemic atrophies, Parkinson’s disease, basal ganglia degeneration, and/or nervous systemic degenerative diseases, PCI = percutaneous coronary intervention. Cox proportional hazards regression analysis was used to determine the hazard ratio of individual variables. (DOCX) [file pone.0259662.s009.docx]

**S5 Table:** Univariable predictors of MI

| **Parameters** | **HR** | **95% CI** | **P value** |
| --- | --- | --- | --- |
| Age, per-1-year increase | 1.01 | 1.01 – 1.02 | 0.002 |
| Female sex | 1.02 | 0.83 – 1.27 | 0.84 |
| **Clinical presentation** |  |  |  |
| Acute coronary syndrome | 3.70 | 2.96 – 4.64 | <0.001 |
| **Comorbidities** |  |  |  |
| Prior myocardial infarction | 1.34 | 0.91 – 1.98 | 0.14 |
| Prior CABG or PCI | 1.00 | 0.69 – 1.44 | 0.99 |
| Heart failure | 2.11 | 1.52 – 2.92 | <0.001 |
| AF/Atrial flutter | 1.94 | 1.42 – 2.64 | <0.001 |
| Stroke | 1.74 | 0.56 – 5.42 | 0.34 |
| Peripheral vascular disease | 0.97 | 0.52 – 1.81 | 0.91 |
| Diabetes | 1.29 | 1.05 – 1.58 | 0.02 |
| Smoker, current or former | 0.91 | 0.75 – 1.10 | 0.32 |
| Chronic kidney disease | 3.12 | 2.25 – 4.32 | <0.001 |
| Chronic lung disease | 1.80 | 1.01 – 3.19 | 0.046 |
| Malignancy | 1.27 | 0.32 – 5.08 | 0.74 |
| Neurodegenerative disease | 0.05 | 0.00 – 881.95 | 0.55 |
| **Procedural data** |  |  |  |
| FFR-guidance | 0.46 | 0.25 – 0.84 | 0.01 |
| Multi-vessel PCI | 1.19 | 0.94 – 1.52 | 0.15 |
| >1 stent to a single vessel | 1.04 | 0.82 – 1.32 | 0.78 |
| **Hospital type** |  |  |  |
| Private hospital | 0.67 | 0.55 – 0.82 | <0.001 |

AF = atrial fibrillation, CABG = coronary artery bypass grafting, CI = confidence interval, FFR = fractional flow reserve, HR = hazard ratio, Neurodegenerative disease = dementia, central nervous systemic atrophies, Parkinson’s disease, basal ganglia degeneration, and/or nervous systemic degenerative diseases, PCI = percutaneous coronary intervention

Cox proportional hazards regression analysis was used to determine the hazard ratio of individual variables.
