# Supplementary material for: A real-world comparison of outcomes between fractional flow reserve-guided versus angiography-guided percutaneous coronary intervention
Source: PLoS One. 2021 Dec 16;16(12):e0259662. doi: 10.1371/journal.pone.0259662 (PMC8675732; doi:10.1371/journal.pone.0259662)
Supplement: S6 Table — AF = atrial fibrillation, CABG = coronary artery bypass grafting, CI = confidence interval, FFR = fractional flow reserve, HR = hazard ratio, Neurodegenerative disease = dementia, central nervous systemic atrophies, Parkinson’s disease, basal ganglia degeneration, and/or nervous systemic degenerative diseases, PCI = percutaneous coronary intervention. Cox proportional hazards regression analysis was used to determine the hazard ratio of individual variables. (DOCX) [file pone.0259662.s010.docx]

**S6 Table:** Multivariable predictors of the primary outcome

| **Parameters** | **HR** | **95% CI** | **P value** |
| --- | --- | --- | --- |
| Age, per-1-year increase | 1.03 | 1.02 – 1.04 | <0.001 |
| Female sex | 0.85 | 0.72 – 0.99 | 0.04 |
| **Clinical presentation** |  |  |  |
| Acute coronary syndrome | 2.76 | 2.31 – 3.30 | <0.001 |
| **Comorbidities** |  |  |  |
| Prior myocardial infarction | 2.14 | 1.64 – 2.80 | <0.001 |
| Prior CABG or PCI | 0.95 | 0.72 – 1.25 | 0.72 |
| Heart failure | 2.05 | 1.67 – 2.52 | <0.001 |
| AF/Atrial flutter | 1.48 | 1.20 – 1.83 | <0.001 |
| Stroke | 2.55 | 1.52 – 4.29 | <0.001 |
| Peripheral vascular disease | 1.48 | 1.08 – 2.05 | 0.02 |
| Diabetes | 1.05 | 0.90 – 1.23 | 0.54 |
| Smoker, current or former | 0.94 | 0.81 – 1.08 | 0.37 |
| Chronic kidney disease | 1.86 | 1.46 – 2.36 | <0.001 |
| Chronic lung disease | 1.62 | 1.16 – 2.25 | 0.004 |
| Malignancy | 3.78 | 2.34 – 6.09 | <0.001 |
| Neurodegenerative disease | 2.33 | 1.04 – 5.23 | 0.04 |
| **Procedural data** |  |  |  |
| FFR-guidance | 0.45 | 0.27 – 0.75 | 0.002 |
| Multi-vessel PCI | 1.31 | 1.09 – 1.56 | 0.004 |
| >1 stent to a single vessel | 1.16 | 0.97 – 1.38 | 0.11 |
| **Hospital type** |  |  |  |
| Private hospital | 1.03 | 0.86 – 1.22 | 0.78 |

AF = atrial fibrillation, CABG = coronary artery bypass grafting, CI = confidence interval, FFR = fractional flow reserve, HR = hazard ratio, Neurodegenerative disease = dementia, central nervous systemic atrophies, Parkinson’s disease, basal ganglia degeneration, and/or nervous systemic degenerative diseases, PCI = percutaneous coronary intervention

Cox proportional hazards regression analysis was used to determine the hazard ratio of individual variables.
