# Supplementary material for: A real-world comparison of outcomes between fractional flow reserve-guided versus angiography-guided percutaneous coronary intervention
Source: PLoS One. 2021 Dec 16;16(12):e0259662. doi: 10.1371/journal.pone.0259662 (PMC8675732; doi:10.1371/journal.pone.0259662)
Supplement: S7 Table — AF = atrial fibrillation, CABG = coronary artery bypass grafting, CI = confidence interval, FFR = fractional flow reserve, HR = hazard ratio, Neurodegenerative disease = dementia, central nervous systemic atrophies, Parkinson’s disease, basal ganglia degeneration, and/or nervous systemic degenerative diseases, PCI = percutaneous coronary intervention. Cox proportional hazards regression analysis was used to determine the hazard ratio of individual variables. (DOCX) [file pone.0259662.s011.docx]

**S7 Table:** Multivariable predictors of all-cause death

| **Parameters** | **HR** | **95% CI** | **P value** |
| --- | --- | --- | --- |
| Age, per-1-year increase | 1.05 | 1.04 – 1.06 | <0.001 |
| Female sex | 0.79 | 0.63 – 0.98 | 0.04 |
| **Clinical presentation** |  |  |  |
| Acute coronary syndrome | 1.47 | 1.16 – 1.87 | 0.002 |
| **Comorbidities** |  |  |  |
| Prior myocardial infarction | 1.64 | 1.16 – 2.33 | 0.01 |
| Prior CABG or PCI | 0.90 | 0.61 – 1.33 | 0.59 |
| Heart failure | 3.11 | 2.41 – 4.02 | <0.001 |
| AF/Atrial flutter | 1.44 | 1.01 – 1.89 | 0.01 |
| Stroke | 3.86 | 2.19 – 6.80 | <0.001 |
| Peripheral vascular disease | 2.53 | 1.75 – 3.66 | <0.001 |
| Diabetes | 0.90 | 0.72 – 1.12 | 0.34 |
| Smoker, current or former | 0.98 | 0.80 – 1.20 | 0.84 |
| Chronic kidney disease | 1.77 | 1.30 – 2.41 | <0.001 |
| Chronic lung disease | 1.74 | 1.18 – 2.56 | 0.01 |
| Malignancy | 5.98 | 3.55 – 10.08 | <0.001 |
| Neurodegenerative disease | 4.45 | 1.96 – 10.08 | <0.001 |
| **Procedural data** |  |  |  |
| FFR-guidance | 0.22 | 0.08 – 0.59 | 0.003 |
| Multi-vessel PCI | 1.36 | 1.05 – 1.74 | 0.02 |
| >1 stent to a single vessel | 1.34 | 1.06 – 1.71 | 0.02 |
| **Hospital type** |  |  |  |
| Private hospital | 0.69 | 0.54 – 0.88 | 0.003 |

AF = atrial fibrillation, CABG = coronary artery bypass grafting, CI = confidence interval, FFR = fractional flow reserve, HR = hazard ratio, Neurodegenerative disease = dementia, central nervous systemic atrophies, Parkinson’s disease, basal ganglia degeneration, and/or nervous systemic degenerative diseases, PCI = percutaneous coronary intervention

Cox proportional hazards regression analysis was used to determine the hazard ratio of individual variables.
