# Supplementary material for: A real-world comparison of outcomes between fractional flow reserve-guided versus angiography-guided percutaneous coronary intervention
Source: PLoS One. 2021 Dec 16;16(12):e0259662. doi: 10.1371/journal.pone.0259662 (PMC8675732; doi:10.1371/journal.pone.0259662)
Supplement: S8 Table — AF = atrial fibrillation, CABG = coronary artery bypass grafting, CI = confidence interval, FFR = fractional flow reserve, HR = hazard ratio, Neurodegenerative disease = dementia, central nervous systemic atrophies, Parkinson’s disease, basal ganglia degeneration, and/or nervous systemic degenerative diseases, PCI = percutaneous coronary intervention. Cox proportional hazards regression analysis was used to determine the hazard ratio of individual variables. (DOCX) [file pone.0259662.s012.docx]

**S8 Table:** Multivariable predictors of CVS death

| **Parameters** | **HR** | **95% CI** | **P value** |
| --- | --- | --- | --- |
| Age, per-1-year increase | 1.03 | 1.02 – 1.04 | <0.001 |
| Female sex | 0.92 | 0.70 – 1.21 | 0.55 |
| **Clinical presentation** |  |  |  |
| Acute coronary syndrome | 1.56 | 1.15 – 2.13 | 0.004 |
| **Comorbidities** |  |  |  |
| Prior myocardial infarction | 1.74 | 1.12 – 2.70 | 0.01 |
| Prior CABG or PCI | 1.06 | 0.66 – 1.71 | 0.81 |
| Heart failure | 4.41 | 3.25 – 5.99 | <0.001 |
| AF/Atrial flutter | 1.66 | 1.19 – 2.30 | 0.003 |
| Stroke | 3.39 | 1.65 – 6.97 | 0.001 |
| Peripheral vascular disease | 1.95 | 1.18 – 3.20 | 0.01 |
| Diabetes | 0.96 | 0.72 – 1.27 | 0.75 |
| Smoker, current or former | 0.80 | 0.61 – 1.03 | 0.09 |
| Chronic kidney disease | 1.44 | 0.96 – 2.16 | 0.08 |
| Chronic lung disease | 1.44 | 0.86 – 2.41 | 0.17 |
| Malignancy | 3.47 | 1.51 – 7.96 | 0.003 |
| Neurodegenerative disease | 3.23 | 1.02 – 10.23 | 0.046 |
| **Procedural data** |  |  |  |
| FFR-guidance | 0.27 | 0.09 – 0.83 | 0.02 |
| Multi-vessel PCI | 1.35 | 0.98 – 1.86 | 0.07 |
| >1 stent to a single vessel | 1.47 | 1.10 – 1.98 | 0.01 |
| **Hospital type** |  |  |  |
| Private hospital | 0.61 | 0.44 – 0.85 | 0.003 |

AF = atrial fibrillation, CABG = coronary artery bypass grafting, CI = confidence interval, FFR = fractional flow reserve, HR = hazard ratio, Neurodegenerative disease = dementia, central nervous systemic atrophies, Parkinson’s disease, basal ganglia degeneration, and/or nervous systemic degenerative diseases, PCI = percutaneous coronary intervention

Cox proportional hazards regression analysis was used to determine the hazard ratio of individual variables.
