# Supplementary material for: A real-world comparison of outcomes between fractional flow reserve-guided versus angiography-guided percutaneous coronary intervention
Source: PLoS One. 2021 Dec 16;16(12):e0259662. doi: 10.1371/journal.pone.0259662 (PMC8675732; doi:10.1371/journal.pone.0259662)
Supplement: S9 Table — AF = atrial fibrillation, CABG = coronary artery bypass grafting, CI = confidence interval, FFR = fractional flow reserve, HR = hazard ratio, Neurodegenerative disease = dementia, central nervous systemic atrophies, Parkinson’s disease, basal ganglia degeneration, and/or nervous systemic degenerative diseases, PCI = percutaneous coronary intervention. Cox proportional hazards regression analysis was used to determine the hazard ratio of individual variables. (DOCX) [file pone.0259662.s013.docx]

**S9 Table:** Multivariable predictors of MI

| **Parameters** | **HR** | **95% CI** | **P value** |
| --- | --- | --- | --- |
| Age, per-1-year increase | 1.01 | 1.01 – 1.02 | 0.001 |
| Female sex | 0.86 | 0.69 – 1.07 | 0.17 |
| **Clinical presentation** |  |  |  |
| Acute coronary syndrome | 4.86 | 3.75 – 6.30 | <0.001 |
| **Comorbidities** |  |  |  |
| Prior myocardial infarction | 2.40 | 1.58 – 3.64 | <0.001 |
| Prior CABG or PCI | 1.05 | 0.72 – 1.52 | 0.81 |
| Heart failure | 1.13 | 0.79 – 1.61 | 0.51 |
| AF/Atrial flutter | 1.45 | 1.05 – 2.01 | 0.03 |
| Stroke | 1.29 | 0.41 – 4.03 | 0.67 |
| Peripheral vascular disease | 0.66 | 0.35 – 1.23 | 0.21 |
| Diabetes | 1.22 | 0.98 – 1.50 | 0.07 |
| Smoker, current or former | 0.90 | 0.74 – 1.09 | 0.28 |
| Chronic kidney disease | 2.13 | 1.50 – 3.02 | <0.001 |
| Chronic lung disease | 1.36 | 0.76 – 2.46 | 0.30 |
| Malignancy | 0.92 | 0.23 – 3.71 | 0.90 |
| Neurodegenerative disease | N/A | N/A | N/A |
| **Procedural data** |  |  |  |
| FFR-guidance | 0.67 | 0.37 – 1.23 | 0.20 |
| Multi-vessel PCI | 1.26 | 0.98 – 1.62 | 0.07 |
| >1 stent to a single vessel | 1.03 | 0.81 – 1.32 | 0.80 |
| **Hospital type** |  |  |  |
| Private hospital | 1.39 | 1.11 – 1.74 | 0.004 |

AF = atrial fibrillation, CABG = coronary artery bypass grafting, CI = confidence interval, FFR = fractional flow reserve, HR = hazard ratio, Neurodegenerative disease = dementia, central nervous systemic atrophies, Parkinson’s disease, basal ganglia degeneration, and/or nervous systemic degenerative diseases, PCI = percutaneous coronary intervention

Cox proportional hazards regression analysis was used to determine the hazard ratio of individual variables.
