# Supplementary material for: A real-world comparison of outcomes between fractional flow reserve-guided versus angiography-guided percutaneous coronary intervention
Source: PLoS One. 2021 Dec 16;16(12):e0259662. doi: 10.1371/journal.pone.0259662 (PMC8675732; doi:10.1371/journal.pone.0259662)
Supplement: S10 Table — Angio = angiography, CABG = coronary artery bypass grafting, FFR = fractional flow reserve, N = number of patients, Neurodegenerative disease = dementia, central nervous systemic atrophies, Parkinson’s disease, basal ganglia degeneration, and/or nervous systemic degenerative diseases, PCI = percutaneous coronary intervention. The baseline characteristics of the two groups were compared using the t-test for continuous variables, and Pearson’s chi square test for dichotomous variables. (DOCX) [file pone.0259662.s014.docx]

**S10 Table:** Baseline characteristics of the stable ischemic heart disease cohort

|  | **Total cohort** | | | **FFR-guided** | **Angio-guided** | **P value** |
| --- | --- | --- | --- | --- | --- | --- |
| **Parameters** | **N=5271** | | | **N=414** | **N=4857** |  |
| Age, years | | 69±11 | | 68±10 | 69±11 | 0.03 |
| Gender, female | | 1354 (26) | | 100 (24) | 1254 (26) | 0.46 |
| **Co-morbid conditions** | |  | |  |  |  |
| Prior myocardial infarction | | 446 (9) | | 18 (4) | 428 (9) | 0.002 |
| Prior PCI / CABG | | | 490 (9) | 43 (10) | 447 (9) | 0.43 |
| Congestive cardiac failure | | | 162 (3) | 12 (3) | 150 (3) | 0.83 |
| Stroke | | | 18 (0) | 1(0) | 17 (0) | 0.72 |
| Peripheral vascular disease | | | 135 (3) | 12 (3) | 123 (3) | 0.65 |
| Atrial fibrillation/flutter | | | 274 (5) | 18 (4) | 256 (5) | 0.42 |
| Diabetes | | | 1362 (26) | 99 (24) | 1263 (26) | 0.35 |
| Smoker, current or former | | | 2022 (38) | 156 (38) | 1866 (38) | 0.77 |
| Malignancy | | | 19 (0) | 1 (0) | 18 (0) | 0.67 |
| Chronic pulmonary disease | | | 82 (2) | 6 (2) | 76 (2) | 0.86 |
| Neurodegenerative disease | | | 8 (0) | 0 (0) | 8 (0) | 0.41 |
| Chronic kidney disease | | | 130 (3) | 3 (1) | 127 (3) | 0.02 |
| **Procedural data** | | |  |  |  |  |
| Single-vessel PCI | | | 4323 (82) | 334 (81) | 3989 (82) | 0.46 |
| Multi-vessel PCI | | | 948 (18) | 80 (19) | 868 (18) |  |
| >1 stent to a single vessel | | | 983 (19) | 54 (13) | 929 (19) | 0.002 |
| **Hospital type** | | |  |  |  |  |
| Public hospital | | | 1772 (34) | 109 (26) | 1663 (34) | 0.001 |
| Private hospital | | | 3499 (66) | 305 (74) | 3194 (66) |  |

Angio = angiography, CABG = coronary artery bypass grafting, FFR = fractional flow reserve, N= number of patients, Neurodegenerative disease = dementia, central nervous systemic atrophies, Parkinson’s disease, basal ganglia degeneration, and/or nervous systemic degenerative diseases, PCI = percutaneous coronary intervention

The baseline characteristics of the two groups were compared using the t-test for continuous variables, and Pearson’s chi square test for dichotomous variables.
