# Supplementary material for: A real-world comparison of outcomes between fractional flow reserve-guided versus angiography-guided percutaneous coronary intervention
Source: PLoS One. 2021 Dec 16;16(12):e0259662. doi: 10.1371/journal.pone.0259662 (PMC8675732; doi:10.1371/journal.pone.0259662)
Supplement: S11 Table — Angio = angiography, CABG = coronary artery bypass grafting, FFR = fractional flow reserve, N = number of patients, Neurodegenerative disease = dementia, central nervous systemic atrophies, Parkinson’s disease, basal ganglia degeneration, and/or nervous systemic degenerative diseases, PCI = percutaneous coronary intervention. The baseline characteristics of the two groups were compared using the t-test for continuous variables, and Pearson’s chi square test for dichotomous variables. (DOCX) [file pone.0259662.s015.docx]

**S11 Table:** Baseline characteristics of the acute coronary syndrome cohort

|  | **Total cohort** | | **FFR-guided** | **Angio-guided** | | **P value** | |
| --- | --- | --- | --- | --- | --- | --- | --- |
| **Parameters** | | **N=5033** | **N=128** | **N=4905** | |  |  |
| Age, years | | 65±13 | 66±13 | 65±13 | 0.72 | |  |
| Gender, female | | 1377 (27) | 39 (31) | 1338 (27) | 0.42 | |  |
| **Co-morbid conditions** | |  |  |  |  | |  |
| Prior myocardial infarction | | 52 (1) | 2 (2) | 50 (1) | 0.55 | |  |
| Prior PCI / CABG | | 246 (5) | 6 (5) | 240 (5) | 0.92 | |  |
| Congestive cardiac failure | | 367 (7) | 13 (10) | 354 (7) | 0.21 | |  |
| Stroke | | 33 (1) | 0 (0) | 33 (1) | 0.35 | |  |
| Peripheral vascular disease | | 123 (2) | 5 (4) | 118 (2) | 0.28 | |  |
| Atrial fibrillation/flutter | | 342 (7) | 12 (9) | 330 (7) | 0.24 | |  |
| Diabetes | | 1278 (25) | 39 (31) | 1239 (25) | 0.18 | |  |
| Smoker, current or former | | 2331 (46) | 70 (55) | 2261 (46) | 0.05 | |  |
| Malignancy | | 24 (1) | 0 (0) | 24 (1) | 0.43 | |  |
| Chronic pulmonary disease | | 89 (2) | 2 (2) | 87 (2) | 0.86 | |  |
| Neurodegenerative disease | | 13 (0) | 0 (0) | 13 (0) | 0.56 | |  |
| Chronic kidney disease | | 223 (4) | 8 (6) | 215 (4) | 0.31 | |  |
| **Procedural data** | |  |  |  |  | |  |
| Single-vessel PCI | | 4303 (86) | 106 (83) | 4197 (86) | 0.38 | |  |
| Multi-vessel PCI | | 730 (15) | 22 (17) | 708 (14) |  |  |  |
| >1 stent to a single vessel | | 946 (19) | 20 (16) | 926 (19) | 0.35 | |  |
| **Hospital type** | |  |  |  |  | |  |
| Public hospital | | 4026 (80) | 104 (81) | 3922 (80) | 0.72 | |  |
| Private hospital | | 1007 (20) | 24 (19) | 983 (20) |  |  |  |

Angio = angiography, CABG = coronary artery bypass grafting, FFR = fractional flow reserve, N= number of patients, Neurodegenerative disease = dementia, central nervous systemic atrophies, Parkinson’s disease, basal ganglia degeneration, and/or nervous systemic degenerative diseases, PCI = percutaneous coronary intervention

The baseline characteristics of the two groups were compared using the t-test for continuous variables, and Pearson’s chi square test for dichotomous variables.
