# Supplementary material for: A real-world comparison of outcomes between fractional flow reserve-guided versus angiography-guided percutaneous coronary intervention
Source: PLoS One. 2021 Dec 16;16(12):e0259662. doi: 10.1371/journal.pone.0259662 (PMC8675732; doi:10.1371/journal.pone.0259662)
Supplement: S12 Table — Angio = angiography, CABG = coronary artery bypass grafting, FFR = fractional flow reserve, N = number of patients, Neurodegenerative disease = dementia, central nervous systemic atrophies, Parkinson’s disease, basal ganglia degeneration, and/or nervous systemic degenerative diseases, PCI = percutaneous coronary intervention. The baseline characteristics of the two groups were compared using the t-test for continuous variables, and Pearson’s chi square test for dichotomous variables. (DOCX) [file pone.0259662.s016.docx]

**S12 Table:** Baseline characteristics of the propensity-matched cohort

|  | **Total cohort** | **FFR-guided** | **Angio-guided** |  |
| --- | --- | --- | --- | --- |
| **Parameters** | **N=1084** | **N=542** | **N=542** | **P value** |
| Age, years | 68±12 | 68±11 | 69±12 | 0.17 |
| Gender, female | 288 (27) | 139 (26) | 149 (28) | 0.49 |
| **Presentation** |  |  |  |  |
| Acute coronary syndrome | 256 (24) | 128 (24) | 128 (24) | 1.00 |
| **Co-morbid conditions** |  |  |  |  |
| Prior myocardial infarction | 40 (4) | 20 (4) | 20 (4) | 1.00 |
| Prior PCI / CABG | 107 (10) | 49 (9) | 58 (11) | 0.08 |
| Congestive cardiac failure | 52 (5) | 25 (5) | 27 (5) | 0.78 |
| Stroke | 4 (0) | 1(0) | 3 (1) | 0.32 |
| Peripheral vascular disease | 38 (4) | 17 (3) | 21 (4) | 0.51 |
| Atrial fibrillation/flutter | 55 (5) | 30 (6) | 25 (5) | 0.49 |
| Diabetes | 277 (26) | 138 (26) | 139 (26) | 0.94 |
| Smoker, current or former | 424 (39) | 226 (42) | 198 (37) | 0.08 |
| Malignancy | 2 (0) | 1 (0) | 1 (0) | 1.00 |
| Chronic pulmonary disease | 14 (2) | 8 (2) | 6 (1) | 0.59 |
| Neurodegenerative disease | 1 (0) | 0 (0) | 1 (0) | 0.32 |
| Chronic kidney disease | 27 (3) | 11 (2) | 16 (3) | 0.33 |
| **Procedural data** |  |  |  |  |
| Single-vessel PCI | 889 (82) | 440 (81) | 449 (83) | 0.48 |
| Multi-vessel PCI | 195 (18) | 102 (19) | 93 (17) |  |
| >1 stent to a single vessel | 148 (14) | 74 (14) | 74 (14) | 1.00 |
| **Hospital type** |  |  |  |  |
| Public hospital | 426 (39) | 213 (39) | 213 (39) | 1.00 |
| Private hospital | 658 (61) | 329 (61) | 329 (61) |  |

Angio = angiography, CABG = coronary artery bypass grafting, FFR = fractional flow reserve, N= number of patients, Neurodegenerative disease = dementia, central nervous systemic atrophies, Parkinson’s disease, basal ganglia degeneration, and/or nervous systemic degenerative diseases, PCI = percutaneous coronary intervention

The baseline characteristics of the two groups were compared using the t-test for continuous variables, and Pearson’s chi square test for dichotomous variables.
